# 4-Thioxo-3,5-dithia-1,7-hepta­nedioic acid

**DOI:** 10.1107/S1600536808030997

**Published:** 2008-09-30

**Authors:** Gui-Sheng Zeng, Jian-Ping Zou, Qiang Peng, Zhen-Hai Wen, Ai-Qing Zhang

**Affiliations:** a School of Environmental and Chemical Engineering, Nanchang Hangkong University, Nanchang, Jiangxi 330063, People’s Republic of China

## Abstract

The complete molecule of the title compound, C_5_H_6_O_4_S_3_, is generated by crystallographic twofold symmetry with the C=S group lying on the rotation axis. The molecules are linked through weak hydrogen-bond contacts by glide-plane operations to form *R*_2_^2^(20) rings and ladder-like *C*(4) chains along the *c* axis.

## Related literature

For related literature, see: Bernstein *et al.* (1995 ▶); El-Bindary *et al.* (1994 ▶); Ng (1995 ▶); Reid (1962 ▶); Strube (1963 ▶).
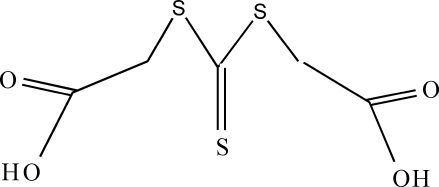


## Experimental

### 

#### Crystal data

C_5_H_6_O_4_S_3_
*M* *_r_* = 226.28
Monoclinic, 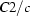

*a* = 18.899 (14) Å
*b* = 5.965 (4) Å
*c* = 7.565 (6) Å
β = 92.992 (2)°
*V* = 851.7 (11) Å^3^
*Z* = 4
Mo *K*α radiation
μ = 0.84 mm^−1^
*T* = 293 (2) K
0.15 × 0.12 × 0.08 mm

#### Data collection

Rigaku Mercury CCD diffractometer
Absorption correction: multi-scan (*CrystalClear*; Rigaku, 2002 ▶) *T*_min_ = 0.912, *T*_max_ = 1.000 (expected range = 0.853–0.935)
2955 measured reflections
967 independent reflections
868 reflections with *I* > 2σ(*I*)
*R*_int_ = 0.021

#### Refinement

*R*[*F* ^2^ > 2σ(*F* ^2^)] = 0.035
*wR*(*F* ^2^) = 0.120
*S* = 1.00
967 reflections
56 parameters
H-atom parameters constrained
Δρ_max_ = 0.45 e Å^−3^
Δρ_min_ = −0.30 e Å^−3^

### 

Data collection: *CrystalClear* (Rigaku, 2002 ▶); cell refinement: *CrystalClear*; data reduction: *CrystalClear*; program(s) used to solve structure: *SHELXTL* (Sheldrick, 2008 ▶); program(s) used to refine structure: *SHELXTL*; molecular graphics: *SHELXTL*; software used to prepare material for publication: *SHELXTL*.

## Supplementary Material

Crystal structure: contains datablocks I, global. DOI: 10.1107/S1600536808030997/si2112sup1.cif

Structure factors: contains datablocks I. DOI: 10.1107/S1600536808030997/si2112Isup2.hkl

Additional supplementary materials:  crystallographic information; 3D view; checkCIF report

## Figures and Tables

**Table 1:** Hydrogen-bond geometry (Å, °)

*D*—H⋯*A*	*D*—H	H⋯*A*	*D*⋯*A*	*D*—H⋯*A*
O1—H1*A*⋯O2^i^	0.82	1.82	2.631 (3)	168

Symmetry code: (i) 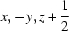.

**Fig. 1.**
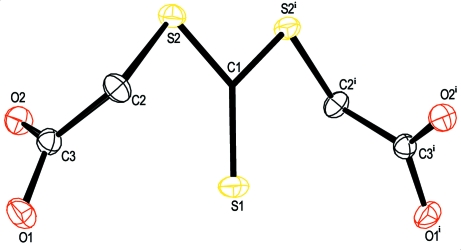
A view of the molecule structure in the title compound, showing 30% displacement ellipsoids for non-H atoms. Symmetry code, i: 1 - x, y, 1/2 - z.

**Fig. 2.**
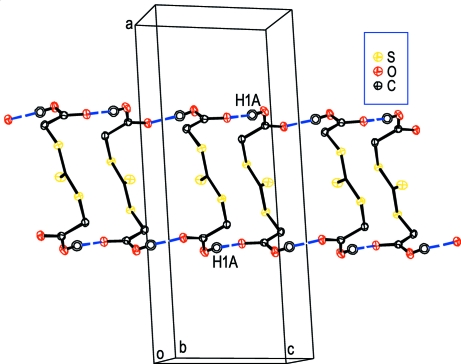
A view of the 1-D chain of the title compound along the c direction. Dashed lines represent the hydrogen bonds.

## supplementary crystallographic information

### Comment

Although the synthesis and the molecular structure of the title compound, also named as trithiocarbodiglycolic acid (TTCD), have been reported, to our knowledge, there is no report on the unit-cell parameters and the crystal structure of TTCD in the literature (Reid, 1962; Strube, 1963; El-Bindary *et al.*, 1994). The crystal structure of a 1:1:1 cocrystal of TTCD and trithiocarbodiglycolate and bis(dicyclohexylammonium) (Ng, 1995) have been reported.

The molecule of the title compound occupies a crystallographic twofold rotation axis with one half-molecule in the asymmetric unit, the C_2_ axis running through the C═S group (Fig. 1). The same molecular symmetry can be observed for the trithiocarbodiglycolate^2-^ and the neutral TTCD molecules in the structure reported by Ng (1995), which crystallises in space group P2/a. Bond distances and angles (Table 1) are close to those in the complexes composed of TTCD reported in the literature (Ng, 1995).

The molecules are linked through weak hydrogen-bond contacts (Table 2) by glide-plane operations to form *R*_2_^2^(20) rings and *C*(4) chains along the *c* axis (Fig. 2).

### Experimental

A mixture of trithiocarbodiglycolic acid (0.25 mmol), CoCl_2_.6H_2_O (0.25 mmol) was dissolved in a 10 ml water in order to synthesize the Co complexes with trithiocarbodiglycolic acid as the ligand. After stirring for about 8 h, the mixed solution was filtered. The filtrate was allowed to stand at room temperature. Colorless crystals of the title complex but not the Co complex with the trithiocarbodiglycolic acid as the ligand were obtained over a period of 10 d.

### Refinement

H atoms were allowed to ride on their respective parent atoms with C—H and O—H distances of 0.97 and 0.82 Å, respectively, and were included in the refinement with isotropic displacement parameters *U*_iso_(H) = 1.2*U*_eq_ (C) and *U*_iso_(H) = 1.5*U*_eq_(O), respectively.

### Crystal data

**Table:**

C_5_H_6_O_4_S_3_	*F*(000) = 464
*M**_r_* = 226.28	*D*_x_ = 1.765 Mg m^−^^3^
Monoclinic, *C*2/*c*	Melting point: not measured K
Hall symbol: -C 2yc	Mo *K*α radiation, λ = 0.71073 Å
*a* = 18.899 (14) Å	Cell parameters from 24 reflections
*b* = 5.965 (4) Å	θ = 3.6–27.4°
*c* = 7.565 (6) Å	µ = 0.84 mm^−^^1^
β = 92.992 (2)°	*T* = 293 K
*V* = 851.7 (11) Å^3^	Prism, colourless
*Z* = 4	0.15 × 0.12 × 0.08 mm

### Data collection

**Table:**

Rigaku Mercury CCD diffractometer	967 independent reflections
Radiation source: rotating-anode generator	868 reflections with *I* > 2σ(*I*)
graphite	*R*_int_ = 0.021
ω scans	θ_max_ = 27.4°, θ_min_ = 3.6°
Absorption correction: multi-scan (CrystalClear; Rigaku, 2002)	*h* = −24→19
*T*_min_ = 0.912, *T*_max_ = 1.000	*k* = −7→7
2955 measured reflections	*l* = −9→9

### Refinement

**Table:**

Refinement on *F*^2^	Primary atom site location: structure-invariant direct methods
Least-squares matrix: full	Secondary atom site location: difference Fourier map
*R*[*F*^2^ > 2σ(*F*^2^)] = 0.035	Hydrogen site location: inferred from neighbouring sites
*wR*(*F*^2^) = 0.120	H-atom parameters constrained
*S* = 1.00	*w* = 1/[σ^2^(*F*_o_^2^) + (0.095*P*)^2^] where *P* = (*F*_o_^2^ + 2*F*_c_^2^)/3
967 reflections	(Δ/σ)_max_ = 0.001
56 parameters	Δρ_max_ = 0.45 e Å^−^^3^
0 restraints	Δρ_min_ = −0.31 e Å^−^^3^

### Special details

**Table:**

Geometry. All e.s.d.'s (except the e.s.d. in the dihedral angle between two l.s. planes) are estimated using the full covariance matrix. The cell e.s.d.'s are taken into account individually in the estimation of e.s.d.'s in distances, angles and torsion angles; correlations between e.s.d.'s in cell parameters are only used when they are defined by crystal symmetry. An approximate (isotropic) treatment of cell e.s.d.'s is used for estimating e.s.d.'s involving l.s. planes.
Refinement. Refinement of *F*^2^ against ALL reflections. The weighted *R*-factor *wR* and goodness of fit *S* are based on *F*^2^, conventional *R*-factors *R* are based on *F*, with *F* set to zero for negative *F*^2^. The threshold expression of *F*^2^ > σ(*F*^2^) is used only for calculating *R*-factors(gt) *etc*. and is not relevant to the choice of reflections for refinement. *R*-factors based on *F*^2^ are statistically about twice as large as those based on *F*, and *R*- factors based on ALL data will be even larger.

### Fractional atomic coordinates and isotropic or equivalent isotropic displacement parameters (Å^2^)

**Table:**

	*x*	*y*	*z*	*U*_iso_*/*U*_eq_	
S1	0.5000	0.03720 (12)	0.2500	0.0393 (3)	
S2	0.43050 (3)	0.48194 (8)	0.31113 (7)	0.0294 (2)	
O1	0.29989 (9)	−0.0371 (2)	0.2569 (2)	0.0384 (4)	
H1A	0.3130	−0.0788	0.3566	0.058*	
O2	0.32991 (7)	0.2197 (2)	0.06832 (17)	0.0343 (4)	
C1	0.5000	0.3111 (4)	0.2500	0.0247 (6)	
C2	0.36648 (10)	0.2799 (3)	0.3739 (2)	0.0301 (5)	
H2A	0.3895	0.1737	0.4552	0.036*	
H2B	0.3300	0.3562	0.4365	0.036*	
C3	0.33171 (9)	0.1520 (3)	0.2204 (2)	0.0282 (4)	

### Atomic displacement parameters (Å^2^)

**Table:**

	*U*^11^	*U*^22^	*U*^33^	*U*^12^	*U*^13^	*U*^23^
S1	0.0395 (5)	0.0202 (4)	0.0581 (6)	0.000	0.0010 (4)	0.000
S2	0.0287 (4)	0.0241 (3)	0.0358 (4)	−0.00075 (17)	0.0046 (2)	−0.00350 (18)
O1	0.0469 (10)	0.0370 (9)	0.0304 (8)	−0.0136 (7)	−0.0073 (7)	0.0040 (6)
O2	0.0414 (8)	0.0352 (8)	0.0260 (8)	−0.0019 (6)	−0.0006 (6)	0.0023 (6)
C1	0.0298 (13)	0.0225 (12)	0.0213 (13)	0.000	−0.0033 (10)	0.000
C2	0.0319 (10)	0.0343 (10)	0.0244 (10)	−0.0068 (8)	0.0033 (7)	−0.0038 (8)
C3	0.0258 (9)	0.0295 (9)	0.0293 (10)	0.0010 (8)	0.0006 (7)	−0.0019 (8)

### Geometric parameters (Å, °)

**Table:**

S1—C1	1.634 (3)	O2—C3	1.219 (2)
S2—C1	1.7438 (18)	C2—C3	1.510 (3)
S2—C2	1.789 (2)	C2—H2A	0.9700
O1—C3	1.314 (2)	C2—H2B	0.9700
O1—H1A	0.8200		
			
C1—S2—C2	101.88 (11)	S2—C2—H2A	108.7
C3—O1—H1A	109.5	C3—C2—H2B	108.7
S1—C1—S2^i^	125.75 (7)	S2—C2—H2B	108.7
S1—C1—S2	125.75 (7)	H2A—C2—H2B	107.6
S2^i^—C1—S2	108.50 (15)	O2—C3—O1	119.50 (18)
C3—C2—S2	114.18 (15)	O2—C3—C2	123.21 (19)
C3—C2—H2A	108.7	O1—C3—C2	117.24 (17)

Symmetry codes: (i) −*x*+1, *y*, −*z*+1/2.

### Hydrogen-bond geometry (Å, °)

**Table:**

*D*—H···*A*	*D*—H	H···*A*	*D*···*A*	*D*—H···*A*
O1—H1A···O2^ii^	0.82	1.82	2.631 (3)	168.

Symmetry codes: (ii) *x*, −*y*, *z*+1/2.
